# New treatments in Alzheimer’s disease

**DOI:** 10.1007/s00415-018-9018-1

**Published:** 2018-08-21

**Authors:** William O. Pickrell, Neil P. Robertson

**Affiliations:** 1 Neurology and Molecular Neuroscience, Swansea University Medical School, Swansea University, Swansea, SA2 8PP UK; 2 Institute of Psychological Medicine and Clinical Neurosciences, Cardiff University, Cardiff, CF14 4XW UK

Alzheimer’s disease is the most common cause of dementia, affecting around 50 million people worldwide. Its prevalence is increasing mainly as a result of an ageing population with considerable associated increases in health and social care costs. As a result, government agencies worldwide have turned funding streams towards unravelling biological mechanisms and developing therapeutic opportunities for the dementias in general. Consequently, a number of novel potential treatments have been developed, although, to date, they have not been shown to significantly alter disease course. In this month’s journal club we look at three large clinical trials of new treatments in Alzheimer’s disease.

## Trial of solanezumab for mild dementia due to Alzheimer’s disease

This paper describes a randomised, controlled, double-blind, multi-centre trial of a monoclonal antibody, solanezumab, in mild Alzheimer’s disease (EXPEDITION 3). Solanezumab is a humanised monoclonal antibody that binds to amyloid beta (Aβ) and increases its clearance. Participants were eligible if they were between 55 and 90 years of age, had probable mild Alzheimer’s disease (mini mental state exam score of between 20 and 26), and had PET or CSF evidence of cerebral beta-amyloid deposition. The primary outcome measure was change in the ADAS-cog14 (Alzheimer’s Disease Assessment Scale 14-item cognitive subscale) score at 80 weeks.

2129 patients were randomised, with 1057 patients receiving 400 mg of solanezumab and 1072 having monthly placebo infusions. There was no significant difference between the treatment and control groups in terms of primary outcome measure. ADAS-cog14 scores changed by 6.65 in the solanezumab group and 7.44 in the placebo group, difference, − 0.80; *P* = 0.10 (higher scores in ADAS-cog14 indicating worsening performance). The trial had a pre-specified hierarchical analysis and so, given the lack of a significant difference between groups in the primary outcome, significance testing was not performed on secondary outcomes. As with other human and animal studies of solanezumab there was a significant reduction in soluble free Aβ concentration (90%) in the treatment arm.

There were no significant differences between the groups in terms of adverse effects and amyloid-related abnormalities on imaging (a concern which had arisen following observations in trials of other monoclonal antibodies that directly target amyloid plaques).

*Comment*. This was a large and well-conducted trial which did not show a significant difference with treatment with an amyloid beta monoclonal antibody. This is now the third randomised control trial of solanezumab and previous trials (EXPEDITION 1 and 2) also did not reach significance in primary endpoints although did show some effect in slowing cognitive decline in secondary analysis. Solanezumab does reduce free Aβ concentrations but this does not seem to translate into clinical benefit.

Honig et al. (2018) N Engl J Med 378:321–330.

## 24-month intervention with a specific multinutrient in people with prodromal Alzheimer’s disease (LipiDiDiet): a randomised, double-blind, controlled trial

This paper describes a randomised, controlled, double-blind trial of a dietary supplement (Fortasyn Connect) in prodromal Alzheimer’s disease (LipiDiDiet). Participants were recruited from memory clinics and were eligible if they were between 55 and 85 years old, had a Mini-Mental State Examination (MMSE) of at least 24 (20 if lower levels of education), and fulfilled criteria for prodromal Alzheimer’s disease including evidence of Alzheimer’s disease pathology in CSF, MRI or PET analysis. Participants were randomised to a drink containing Fortasyn Connect (a combination of docosahexaenoic and eicosapentaenoic acid, uridine monophosphate, choline, vitamins, phospholipids, and selenium) or a placebo. This specific combination is designed to provide neuroprotection by providing compounds for brain phospholipid synthesis and addressing multiple Alzheimer’s disease-related processes. The primary outcome was the change in a composite score of a neuropsychological test battery over the 2 years of the trial period. The neuropsychological test battery included tests of immediate recall, delayed recall, word recognition and category fluency.

311 patients were randomised (mean age 71, 50% male) with 153 in the treatment group and 158 in the placebo group. 59 (37%) participants in the control group and 62 (41%) participants in the treatment group were diagnosed with dementia during the trial. There was no significant difference between the groups in the primary end point. There were no significant differences in four of the secondary end points (changes in MRI whole brain volume, memory domain, executive function domain and total neuropsychological battery score). The treatment did show benefit in three of the secondary outcomes: there was a slower hippocampal atrophy rate, a smaller increase in ventricular volume and less worsening in the clinical dementia rating-sum of boxes scores in the treatment group.

*Comment*. This was a well-conducted trial and the first published trial of a non-pharmacological intervention in prodromal Alzheimer’s disease. A dietary supplement seems an attractive option as a treatment for Alzheimer’s disease compared with powerful pharmacological agents, but this trial does not provide enough evidence for use of Fortasyn Connect in prodromal Alzheimer’s disease.

Soininen et al. (2017) Lancet Neurol. 16:965–75.

## Randomized trial of verubecestat for mild-to-moderate Alzheimer’s disease

This paper describes a randomised, double-blind, multi-centre trial of verubecestat in mild and moderate Alzheimer’s disease. Verubecestat inhibits β-site APP–cleaving enzyme 1 (BACE-1; ) and therefore prevents the cleavage of amyloid precursor protein to Aβ. Participants were eligible if they were between 55 and 85 years old, had probable Alzheimer’s disease, and a mini mental state examination score of between 15 and 26. The primary outcomes were changes in scores in the cognitive sub-scale of the Alzheimer’s disease Assessment Scale (ADAS-cog) and Alzheimer’s Disease Cooperative Study Activities of Daily Living Inventory scale (ADCS-ADL) (a measure of day to day function).

1958 patients were randomised; 653 received 12 mg of verubecestat daily, 652 received 40 mg of verubecestat daily and 653 received placebo. The trial was terminated 5 months prematurely due to futility—there were no significant differences between the verubecestat groups and placebo in terms of primary outcomes. The verubecestat groups did have a significant reduction in CSF A − 40, A − 42 and sAPPβ levels when compared with the placebo group. There were more adverse events in the treatment groups. Specific adverse events which were more common in the treatment group included falls, rash, weight decrease, hair colour change and suicidal ideation.

*Comment*. Another large and well-constructed trial which did not reach significance in its primary outcomes. It seems that the drug is effective in reducing CSF levels of Aβ but this does not translate into clinical benefit. There seemed to be more adverse effects with this drug when compared with other treatments.

Egan et al. (2018) N Engl J Med. 378:1691–703.

## Conclusion

The three papers we have reviewed in this journal club represent large, well-constructed, randomised controlled trials in new and promising treatments for Alzheimer’s disease. Despite all three treatments having significant pre-clinical and clinical evidence for their suitability in treating Alzheimer’s disease, all three failed to reach their primary outcomes. Although disappointing for the researchers and patients with Alzheimer’s disease, it is important that lessons can be learnt from these ‘negative’ results, in particular in the evolution of trial design in this discipline, and it is refreshing for the methodology and evidence to be presented in high-impact journals.

There are several potential reasons for the failure of these treatments to show significant clinical effects, the most important being perhaps that participants are too far into their disease course to benefit from these treatments by the time they have started the trials. This might mean that targeting preventative treatments at a population level is a more sensible approach. However, efforts to develop a disease-modifying treatment remains a priority for healthcare providers worldwide, as effective intervention would have the potential to transform the lives of millions of people with Alzheimer’s disease and their carers who are affected by the debilitating consequences of this disease.

